# Clinical utility of ambulatory ECG monitoring and 2D-ventricular strain for evaluation of post-COVID-19 ventricular arrhythmia

**DOI:** 10.1186/s12872-024-03982-0

**Published:** 2024-08-16

**Authors:** Rehab M. Hamdy, Mohammed Samy, Huda Shaban Mohamed

**Affiliations:** 1 Present Address: Department of Cardiology, Faculty of Medicine (for Girls), Al-Azhar University, Cairo, Egypt; 2 Department of Cardiology, Faculty of Medicine (for Boys), Al-Azhar University, Cairo, Egypt

**Keywords:** COVID-19, Premature ventricular contractions, rMSSD, LV-global longitudinal strain, RV-global longitudinal strain

## Abstract

**Background:**

A relatively common complication of COVID -19 infection is arrhythmia. There is limited information about myocardial deformation and heart rate variability (HRV) in symptomatic post COVID patients presented by ventricular arrhythmia.

**Aim of the study:**

Our goal was to assess 2D-ventricular strain and heart rate variability indices (evaluated by ambulatory ECG monitoring) in post-COVID-19 patients suffering from ventricular arrhythmia.

**Methods:**

The current observational case–control study performed on 60 patients one month after they had recovered from the COVID-19 infection. Thirty healthy volunteers served as the control group. Each participant had a full medical history review, blood tests, a 12-lead surface electrocardiogram (ECG), 24-h ambulatory ECG monitoring, and an echo-Doppler examination to evaluate the left ventricular (LV) dimensions, tissue Doppler velocities, and 2D-speckle tracking echocardiography (2D-STE) for both the LV and right ventricular (RV) strain.

**Results:**

Symptomatic post-COVID patients with monomorphic premature ventricular contractions (PVCs) showed a substantial impairment of LV/RV systolic and diastolic functions, LV/RV myocardial performance (MPI) with reduced indices of HRV. Patients with higher versus lower ventricular burden had poorer functional status, higher levels of inflammatory biomarkers and reduced parameters of HRV (New York Heart Association (NYHA) class: 2.1 ± 0.9 vs. 1.5 ± 0.6, *p* < 0.001, C-reactive protein (CRP): 13.3 ± 4.1 vs. 8.3 ± 5.9 mg/L, *p* < 0.0001, low frequency/high frequency (LF/HF): 3.6 ± 2.4 vs. 2.2 ± 1.2, *p* < 0.002, the root mean square of the difference between successive normal intervals (rMSSD): 21.8 ± 4.7 vs. 29.3 ± 14.9 ms, *p* < 0.039 and the standard deviation of the RR interval (SDNN): 69.8 ± 19.1 vs.108.8 ± 37.4 ms, *p* < 0.0001). The ventricular burden positively correlated with neutrophil/lymphocyte ratio (NLR) (r = 0.33, *p* < 0.001), CRP (*r* = 0.60, *p* < 0.0001), while it negatively correlated with LV-global longitudinal strain (GLS) (*r* = -0.38, *p* < 0.0001), and RV-GLS (*r* = -0.37, *p* < 0.0001).

**Conclusions:**

Patients with post-COVID symptoms presented by ventricular arrhythmia had poor functional status. Patients with post-COVID symptoms and ventricular arrhythmia had subclinical myocardial damage, evidenced by speckle tracking echocardiography while having apparently preserved LV systolic function. The burden of ventricular arrhythmia in post-COVID patients significantly correlated with increased inflammatory biomarkers and reduced biventricular strain.

**Supplementary Information:**

The online version contains supplementary material available at 10.1186/s12872-024-03982-0.

## Background

COVID-19 infection is characterized by significant respiratory pathology as well as a number of extrapulmonary symptoms, including thrombotic problems, cardiac dysfunction, and arrhythmia [1]. One of the main SARS-COV consequences during the China outbreak was cardiac arrhythmia [2]. The rate of ventricular arrhythmia was nearly doubled in patients with increased troponin T levels at intensive care unit (ICU) admission, occurring in 7% of patients [3]. However, the precise pathophysiology causing COVID-19's ventricular arrhythmia may be complex and difficult to determine.

The post-COVID condition is becoming a significant public health concern. A number of national and international research programs promise to clarify the complexity of this disease, but the current understanding of pathophysiological mechanisms is still limited [4]*.*

## Methods

### Aim of the study

Our goal was to assess 2D-ventricular strain and heart rate variability (defined by ambulatory ECG monitoring) in symptomatic post- COVID-19 patients presented by ventricular arrhythmias. Between October 2021 and November 2022, this observational case–control study was conducted at the cardiology outpatient clinic of Al-Zahra University Hospital. We enrolled 60 consecutive patients who recovered from active COVID-19 infection between one month and 6 months with preserved left ventricular systolic function (LVEF > 55%) during their follow-up visits due to persistence of symptoms. All included patients had persistent palpitation ± dyspnea [using NYHA and post-COVID functional status (PCFS)] with documented monomorphic premature ventricular contractions (PVCs) in their resting ECG (in whom PVCs could not be appreciated in their initial evaluation during active COVID-19 infection either by history, ECG or Holter recording). Since certain PVCs may be fusion rather than polymorphic PVCs, the morphology of the PVCs was established based on resting ECG and > 98% of comparable morphology in ECG monitoring analysis. Control group have been recruited via digital survey during vaccine campaign and information submitted over a phone conversation or over an online link. All participants were chosen irrespective of their social standing, education level, and gender. Thirty- age and sex matched healthy individuals with no history of previous COVID infection confirmed by negative reverse transcription polymerase chain reaction on a nasopharyngeal swab) have been included.

We excluded patients with known cardiomyopathies, ischemic heart disease, significant valvular disease, congenital heart disease, pulmonary hypertension, advanced renal and liver diseases, atrial fibrillation, previous documented ventricular arrhythmias or previous ablation, polymorphic ventricular arrhythmia and patients who received COVID vaccinations.

All studied cases were subjected to a detailed history with special emphasis on current and previous symptoms (representing "arrhythmic" symptoms as palpitations or syncope), diagnostic tests, and medications during COVID-19. Laboratory investigation analyses were performed to assess white blood cells (WBCs), neutrophil/lymphocyte ratio (NLR), erythrocytic sedimentation rate (ESR), C-reactive protein (CRP), as well as O2 saturation. Five days after the onset of COVID symptoms, all patients underwent CT chest scans to rule out chest involvement. A CT scan was repeated prior to trial enrollment.

*12-leads surface ECG* for localization of ventricular arrhythmia (morphology, axis, precordial transition, and suggested localization) [5] and [6] and measurement of QTc interval.

*24-h ambulatory ECG monitoring* was performed at least one month after recovery from COVID-19 for evaluation of burden and morphology of ventricular arrhythmias in addition to assessment of heart rate variability parameters [7].

- Holter Windows 5.1 with software Version 1 (SEER Light, GE Healthcare, Milwaukee, WI, US) was used for data acquisition and analysis. A 3-channel ECG was obtained and analyzed for the whole period of 24 h. The HRV parameters were evaluated for 24 h. Parameters were investigated in two separate periods: daytime (8:00–12:00 a.m.) and nighttime (12:00–8:00 a.m.). Time-domain and frequency-domain indices were used to evaluate HRV.

The following time domain parameters were measured [8]:

- Mean RR (ms) = the mean of the RR interval.
- pNN50 (%) = the percentage of differences greater than 50 ms between successive normal RR intervals in a 24-h ECG record.
- SDNN (ms) = the standard deviation of the RR interval.
- rMSSD (ms) = the root mean square of the difference between successive normal intervals.

The following frequency domain parameters were measured [8]:

- LF (nu) = it includes the absolute power of the low-frequency band range between 0.04 Hz and 0.15 Hz.
- HF (nu) = it includes the absolute power of the high-frequency band range between 0.16 Hz and 0.4 Hz.
- LF/HF ratio = the ratio of LF-to-HF power. It reflects the sympathovagal balance and can be used to estimate HRV in general [9].

*A Conventional transthoracic echo-Doppler study* was performed for all cases using Vivid-E9 GE system (GE Ultrasound; Horten, Norway) with a multi-frequency (2.5 MHz) matrix probe M3S, and simultaneous ECG physio-recording signals were displayed with all recorded echocardiographic images and loops. Offline analysis was performed using EchoPAC. GE version 110–1.3. All parameters were taken according to the guidelines of the American Society of Echocardiography [10]. LV dimensions, volumes, ejection fraction (EF), left atrial (LA) diameter, LA volume index (LAVI), E and A velocities and E/A ratio for both mitral and tricuspid valve flow were assessed. Tissue Doppler Imaging (TDI) for assessment of mitral and tricuspid annular systolic and diastolic velocities obtained [11] in addition to the E/Eʼ ratio [12]. The following TDI parameters were assessed; LV AV. S'_4_ by averaging systolic 4-sites mitral annular velocities by TDI, LV AV. E'_4_ by averaging early diastolic 4-mitral annular velocities by TDI, LV E/ AV.E'_4_ which is the ratio of early diastolic mitral valve velocity to the average early diastolic 4-mitral annular velocities by TDI. Mitral annulus plane systolic excursion (MAPSE)***,*** tricuspid annulus plane systolic excursion (TAPSE), LV myocardial performance index (LV-MPI) and RV myocardial performance index (RV-MPI) were assessed [10].

*2D speckle tracking echocardiography* was used to assess LV global longitudinal strain (LV-GLS), RV-GLS, and RV-free wall strain (RV_FW_).

We classified post-COVID patients according to the ventricular burden into:

- Group Ia: included 36-post-COVID patients with ventricular burden ≤ 10%.
- Group Ib: included 24-post-COVID patients with ventricular burden > 10%.

### Statistical analysis

We checked all the parameters using the Shapiro–Wilk test to assess if they were normally distributed. Normally distributed variables were reported as mean ± standard deviation (SD), and the groups were compared using ANOVA, paired or unpaired t-tests, as appropriate. Non- normally distributed variables were reported as median (interquartile range [IQR]), and the groups were compared using the Mann–Whitney test or Kruskal Wallis test, as appropriate. Using the appropriate Fisher's exact test or Chi-squared test, the categorical variables were compared as frequencies (percentages). To assess the association between variables, Pearson and Spearman correlation analyses were performed. For detecting the association between the occurrence of ventricular arrhythmia (high versus low burden) in symptomatic post COVID-19 patients, multiple regression and binary logistic regression analyses were conducted. Results of logistic regression were reported as odds ratio (OR) with 95% confidence interval (CI). To evaluate the individual effects of various confounding factors, we constructed number of multivariate regression models. To determine an appropriate theoretical model that matches the data collected, the variables in the multivariable logistic regression analysis were approved using a repeated backward-stepwise approach (inclusion criteria *p* < 0.05, exclusion criteria *p* > 0.10) in the univariable logistic regression analysis. *P*-value < 0.05 considered statistically significant. The SPSS 23.0 software (SPSS Inc., Chicago, IL, USA) was used to measure all statistics.

## Results

Table 1 showed the comparison between post-COVID (Ia and Ib) and control groups according to the demographic and laboratory data.

**Table 1:** Comparison between post-COVID (Ia and Ib) and control groups regarding the demographic and laboratory data

Variables	Group Ia	Group Ib	Control	*P*^a^	*P*^b^	*P*^c^
	*N* = 36	*N* = 24	*N* = 30			
**Age (years) (mean ± SD)**	41.1 ± 9.2	42.5 ± 13.6	38.0 ± 9.9	0.87	0.46	0.27
**Sex (no &%)**						
** • Male**	11 (30.5%)	10 (41.7%)	8 (26.7%)	0.48		
** • Female**	25 (69.5%)	14 (58.3%)	22 (73.3%)			
**HTN (no &%)**	4 (11.1%)	8 (33.3%)	-	0.015	-	-
**DM (no &%)**	6 (16.7%)	6 (25%)	-	0.43	-	-
**Smoking (no &%)**	4 (11.1%)	6 (25%)	-	0.16	-	-
**Weight (Kg) (mean ± SD)**	81.6 ± 9.6	81.8 ± 9.9	76.7 ± 11.4	0.99	0.13	0.18
**Height (cm) (mean ± SD)**	166.5 ± 5.8	166.5 ± 5.3	165.9 ± 6.7	0.99	0.92	0.92
**BSA (m**^**2**^**) (mean ± SD)**	1.9 ± 0.1	1.9 ± 0.1	1.9 ± 0.2	1.00	0.17	0.20
**BMI (Kg/m**^**2**^**) (mean ± SD)**	29.6 ± 4.2	29.5 ± 3.6	27.8 ± 3.5	1.00	0.17	0.26
**Post-COVID duration (months)**	3.3 ± 1.8	3.3 ± 2.0	-	0.87	-	-
**Dsypnea**	17 (42.7%)	13 (54.2%)	-		-	-
**NYHA class (mean ± SD)**	1.5 ± 0.6	2.1 ± 0.9	0.00 ± 0.0	0.001	0.0001	0.0001
**PCFS grade (mean ± SD)**	1.5 ± 0.6	2.1 ± 0.9	0.00 ± 0.0	0.0001	0.0001	0.0001
**Hospital admission during active COVID infection**	2 (5.6%)	3 (12.5%)		0.34	-	-
**Home isolation during active COVID infection**	34 (94.4%)	21 (87.5%)		0.14	-	-
**Results of CT during acute stage (no &%)**						
** • Normal**	34 (94.4%)	21 (87.5%)	-	0.14	-	-
** • Abnormal**	2 (5.6%)	3 (12.5%)	**-**	0.34	**-**	**-**
**WBCs (× 10**^**3**^**/cmm) (mean ± SD)**	7.6 ± 2.1	8.1 ± 2.5	6.4 ± 1.4	0.65	0.045	0.008
**NLR (mean ± SD)**	2.1 ± 0.6	2.2 ± 0.7	1.4 ± 0.4	0.65	0.0001	0.0001
**Hb% (gm/dL) (mean ± SD)**	11.9 ± 1.0	11.8 ± 0.1	12.5 ± 0.7	0.93	0.017	0.014
**ESR (1st hour) (mean ± SD)**	15.5 ± 7.6	14.9 ± 7.2	13.8 ± 7.9	0.97	0.65	0.86
**CRP (mg/L) (mean ± SD)**	8.3 ± 5.9	13.3 ± 4.1	4.5 ± 2.2	0.0001	0.003	0.0001
**O2 sat (%)(mean ± SD)**	97.8 ± 3.2	96.7 ± 2.6	99.3 ± 0.7	0.15	0.07	0.001

*Abbreviations*:* P*^a^ = group Ia vs. Ib, *P*^b^ = group Ia vs. control, *P*^c^ = group Ib vs. control, *HTN* Hypertension, *DM* Diabetes millets, *BSA* Body surface area, *BMI* Body mass index, *NYHA* New York Heart Association, *PCFS* Post-COVID functional status, *NLR* Neutrophil/lymphocyte ratio, *ESR* Erythrocytic sedimentation rate, *CRP* C-reactive protein, *O2 sat* Oxygen saturation

Table 2 demonstrated that group Ib had significantly higher resting HR, while the remaining parameters did not differ between both groups. The possible origins of PVCs were right ventricular outflow tract (RVOT) origin in 70%, left ventricular outflow tract (LVOT) origin in 21.7% and non-outflow origin in 8.3% of symptomatic post-COVID patients.

**Table 2:** Comparison between post-COVID groups (Ia and Ib) regarding the ECG characterization and localization of PVCs

Variables	Group Ia	Group Ib	*P*
	*N* = 36	*N* = 24	
**Resting HR (bpm)**	82.5 ± 12.4	91.0 ± 10.7	0.006
**Precordial transition**			
** ≤ V2**	10 (27.8%)	8 (33.3%)	0.65
** ≥ V3**	26 (72.8%)	16 (66.7%)	
**PVC morphology**			
• **LBBB**	34 (94.4%)	21 (87.5%)	0.34
• **RBBB**	2 (5.6%)	3 (12.5%)	
**PVC axis**			
• **LAD**	1 (2.8%)	-	0.41
• **RAD**	35 (97.2%)	24 (100%)	
**Possible PVC origin**			
• **RVOT**	26 (72.2%)	16 (66.7%)	0.64
• **LVOT**	8 (22.2%)	5 (20.8%)	
• **Non-outflow**	2 (5.6%)	3 (12.5%)	
**QTc interval using Bazett formula (ms) (mean ± SD)**	441.8 ± 16.5	451.4 ± 18.6	0.05
**QTc interval using Fridericia formula (ms) (mean ± SD)**	436.2 ± 15.8	442.4 ± 17.9	0.24

*Abbreviations*:* PVC* Premature ventricular contraction, *LBBB* Left bundle branch block, *RBBB* Right bundle branch block, *LAD* Left axis deviations, *RAD* Right axis deviation, *RVOT* Right ventricular outflow tract, *LVOT* Left ventricular outflow tract

Post-COVID Ia and Ib groups had significantly lower LVEF compared to control, despite that the fact that all LVEF were within the normal range. Meanwhile, group Ia and group Ib showed significant reductions in LV AV. S'_4_, LV-GLS, MAPSE, RV-S'_Lat_, RV-GLS, RV-GLS_FW_, and TAPSE compared to control. Post COVID patients had significantly reduced LV-GLS regarding control (16.2 ± 2.8% vs. 20.2 ± 2.0% respectively with *p* < 0.0001). In addition, post-COVID Ia and Ib groups had significant increase of LA diameter, LAVI, E/A, LV E/AV.E'_4_, LV-MPI, and RV-MPI compared to control (Table 3).

**Table 3:** Comparison among post-COVID (Ia and Ib) and control groups regarding the echo-Doppler parameters

Variables	Group Ia	Group Ib	Control	*P*^a^	*P*^b^	*P*^c^
	*N* = 36	*N* = 24	*N* = 30			
LVEDD (mm) (mean ± SD)	49.9 ± 6.0	51.9 ± 3.5	47.5 ± 3.7	0.28	0.09	0.003
LVESD (mm)(mean ± SD)	31.5 ± 3.6	33.0 ± 2.9	29.6 ± 3.6	0.20	0.07	0.001
IVS (mm) (mean ± SD)	8.9 ± 1.0	9.2 ± 1.7	8.5 ± 1.4	0.59	0.38	0.09
LVPW (mm) (mean ± SD)	8.7 ± 1.5	9.0 ± 1.7	8.6 ± 0.9	0.71	0.98	0.62
EF% (M-mode) (mean ± SD)	65.4 ± 2.5	63.1 ± 3.4	68.0 ± 6.1	0.12	0.04	0.0001
LA diameter (mm)(mean ± SD)	35.7 ± 4.1	38.0 ± 5.4	32.2 ± 4.2	0.14	0.006	0.0001
LAVI (ml/m^2^)(mean ± SD)	26.3 ± 13.3	28.3 ± 10.4	19.7 ± 3.1	0.72	0.027	0.007
LVEDV (ml) (mean SD)	112.1 ± 42.1	121.0 ± 26.4	86.9 ± 22.8	0.56	0.007	0.001
LVESV (ml) (mean ± SD)	39.0 ± 14.7	43.3 ± 9.5	30.6 ± 7.5	0.33	0.011	0.0001
EF% (bi-plane) (mean ± SD)	66.1 ± 5.5	64.3 ± 3.3	67.9 ± 3.6	0.25	0.24	0.01
E/A (mean ± SD)	1.2 ± 0.3	1.3 ± 0.4	1.6 ± 0.4	0.47	0.0001	0.007
LV AV. S'_4_ (cm/s) (mean ± SD)	6.1 ± 1.3	5.9 ± 1.2	7.4 ± 1.6	0.75	0.001	0.0001
LV AV.E' _4_ (cm/s) (mean ± SD)	7.9 ± 2.7	7.3 ± 1.7	10.3 ± 1.5	0.56	0.0001	0.0001
LV E/ AV.E' _4_ (mean ± SD)	10.5 ± 3.5	11.8 ± 3.6	6.9 ± 1.3	0.26	0.0001	0.0001
MAPSE (mm) (mean ± SD)	12.8 ± 1.9	9.3 ± 1.8	17.3 ± 2.8	0.0001	0.0001	0.0001
LV-MPI (mean ± SD)	0.47 ± 0.1	0.61 ± 0.3	0.30 ± 0.2	0.018	0.001	0.0001
LV-GLS (%)(mean ± SD)	16.6 ± 2.8	15.7 ± 2.9	20.2 ± 2.0	0.44	0.0001	0.0001
TAPSE (mm) (mean ± SD)	17.1 ± 4.3	13.2 ± 3.4	21.7 ± 3.3	0.0002	0.0001	0.0001
RV-MPI (mean ± SD)	0.41 ± 0.2	0.53 ± 0.2	0.19 ± 0.1	0.031	0.0001	0.0001
RV-S'_Lat_ (cm/s) (mean ± SD)	9.6 ± 1.7	8.8 ± 1.0	12.8 ± 2.1	0.95	0.035	0.032
RV-GLS (%)(mean ± SD)	16.1 ± 4.7	15.4 ± 3.6	22.1 ± 1.8	0.73	0.0001	0.0001
RV-GLS_FW_ (%)(mean ± SD)	16.5 ± 3.8	14.8 ± 4.2	24.6 ± 2.9	0.20	0.0001	0.0001

*Abbreviations*:* P*^a^ group Ia vs. Ib, *P*^b^ group Ia vs. control, *P*^c^ group Ib vs. control, *LVEDD* LV end diastolic dimensions, *LVESD* LV end systolic dimensions, *IVSd* Interventricular septum in diastole,* LVPWd* LV posterior wall in diastole, *LVEDV* LV end diastolic volume, *LVESV* LV end systolic volume, *LV AV. S'4* average systolic 4-sites mitral annular velocities by tissue Doppler imaging, *LV AV.E' 4* average early diastolic 4-mitral annular velocities by TDI, *LV E/ AV.E' 4* ratio of early diastolic mitral valve velocity/average early diastolic 4-mitral annular velocities by TDI, *MAPSE* mitral annulus plane systolic excursion, *LV-MPI* LV myocardial performance, *LV-GLS* LV global longitudinal strain, *TAPSE* Tricuspid annulus plane systolic excursion, *RV-MPI* RV myocardial performance index, *RV-S'Lat* RV myocardial systolic velocity at the RV lateral wall by TDI, *RV-GLS* RV global longitudinal strain, *RV-GLS*_FW_ RV free wall strain

In the current study, group Ia and group Ib had significant increases in minimum HR, average HR, % of tachycardia, % of ventricular arrhythmia and LF/HF compared to control. Moreover, LF, HF, pNN50, rMSSD, and SDNN were significantly lower in group Ia and group Ib compared to control (Table 4).

**Table 4:** Comparison among post-COVID (Ia and Ib) and control groups regarding 24-h Holter parameters

Variables	Group Ia *N* = 36	Group Ib *N* = 24	Control *N* = 30	*P*^a^	*P*^b^	*P*^c^
Minimum HR (bpm) (mean ± SD)	55.7 ± 10.5	61.1 ± 5.4	51.1 ± 6.6	0.035	0.06	0.0001
Maximum HR (bpm) (mean ± SD)	142.1 ± 20.9	148.9 ± 17.3	139.0 ± 26.1	0.46	0.84	0.23
Average HR (bpm) (mean ± SD)	81.7 ± 12.7	86.2 ± 9.2	70.4 ± 6.6	0.22	0.0001	0.0001
% of tachycardia (median, IQR)	18.5 (12.0)	25.5 (23.0)	3.5 (7.0)	0.0001		
% of bradycardia (median, IQR)	1.0 (6.5)	1.0 (1.8)	2.5 (7.0)	0.12		
% of ventricular arrhythmia (mean ± SD)	3.0 (5.2)	19.0 (8.5)	0.0	0.0001		
No. of isolated PVCs (median, IQR)	1364.0 (1977)	16,782.5 (14416.0)	0.0	0.0001		
No. of bigeminy (median, IQR)	0.0 (14.8)	1743.0 (11146.7)	0.0	0.0001		
No. of non-sustained VT runs (median, IQR)	0.0 (0.0)	0.0 (17.5)	0.0	0.0001		
No. of couplets (median, IQR)	0.0 (2.0)	90.0 (278.5)	0.0	0.0001		
LF/HF (mean ± SD)	2.2 ± 1.2	3.6 ± 2.4	1.4 ± 0.4	0.002	0.06	0.0001
HF (nu) (mean ± SD)	13.5 ± 5.6	10.0 ± 4.2	32.9 ± 7.5	0.01	0.0001	0.0001
LF (nu) (mean ± SD)	24.2 ± 7.0	23.0 ± 4.5	46.1 ± 8.4	0.40	0.0001	0.0001
pNN50% (median, IQR)	6.1 (11.6)	4.8 (5.9)	11.2 (9.7)	0.17	0.01	0.0001
rMSSD (ms) (mean ± SD)	29.3 ± 14.9	21.8 ± 4.7	38.1 ± 10.4	0.039	0.006	0.0001
SDNN (ms) (mean ± SD)	108.8 ± 37.4	69.8 ± 19.1	148.9 ± 34.3	0.0001	0.0001	0.0001

*Abbreviations*:* P*^a^ group Ia vs. Ib, *P*^b^ group Ia vs. control, *P*^c^ group Ib vs. control, *HR* Heart rate, *LF/HF* the ratio of low-frequency/high-frequency power, *LF* Low frequency, *HF* High frequency, *rMSSD* root mean square of the difference between successive normal intervals, *pNN50* the percentage of the number of pairs of consecutive beat-to-beat intervals that differed by 50 ms, *SDNN* the standard deviation of the RR interval, *SD* Standard deviation, *IQR* Interquartile range

### Correlation between ventricular burden and functional status, laboratory investigations, different Holter and echo-Doppler indices among the studied population

The ventricular burden positively correlated with NYHA class, PCFS, WBCs, NLR, CRP, and LF/HF among our studied population. While there were negative correlations between the ventricular burden with O2 sat., HF, LF, p NN50, rMSSD and SDNN (Table 5).

**Table 5:** Correlation between ventricular burden and functional status, laboratory investigations, ambulatory ECG monitoring and echo-Doppler parameters

Variables	R	P
Functional status and laboratory investigations		
Age	0.12	**0.20**
HTN	0.46	**0.0001**
BMI	0.16	**0.13**
NYHA class	0.59	**0.0001**
PCFS grade	0.59	**0.0001**
WBCs (× 10^3^/cmm)	0.23	**0.038**
NLR	0.33	**0.001**
CRP (mg/L)	0.60	**0.0001**
O2 sat. (%)	-0.36	**0.0001**
Resting ECG		
HR (bmp)	0.14	**0.0001**
Selected ambulatory ECG monitoring		
Av. HR (bpm)	0.34	**0.001**
LF/HF	0.26	**0.02**
HF (nu)	-0.46	**0.0001**
LF (nu)	-0.41	**0.0001**
pNN50%	-0.39	**0.0001**
rMSSD (ms)	-0.42	**0.0001**
SDNN (ms)	-0.563	**0.0001**
Selected echo-parameters		
LAVI (ml/m^2^)	0.25	**0.02**
LVEDD (mm)	0.31	**0.003**
LVESD (mm)	0.34	**0.001**
LVEDV (ml)	0.27	**0.01**
LVESV (ml)	0.26	**0.013**
EF (biplane) (%)	-0.26	**0.015**
LV AV. S'_4_ (cm/s)	-0.27	**0.009**
LV E/ AV.E' _4_	0.45	**0.0001**
MAPSE (mm)	-0.60	**0.0001**
LV-MPI	0.42	**0.0001**
LV-GLS (%)	-0.38	**0.0001**
TAPSE (mm)	-0.53	**0.0001**
RV-MPI	0.45	**0.0001**
RV-S'_Lat_ (cm/s)	-0.49	**0.0001**
RV-GLS (%)	-0.37	**0.0001**
RV-GLS_FW_ (%)	-0.52	**0.0001**

**Fig. 1:**
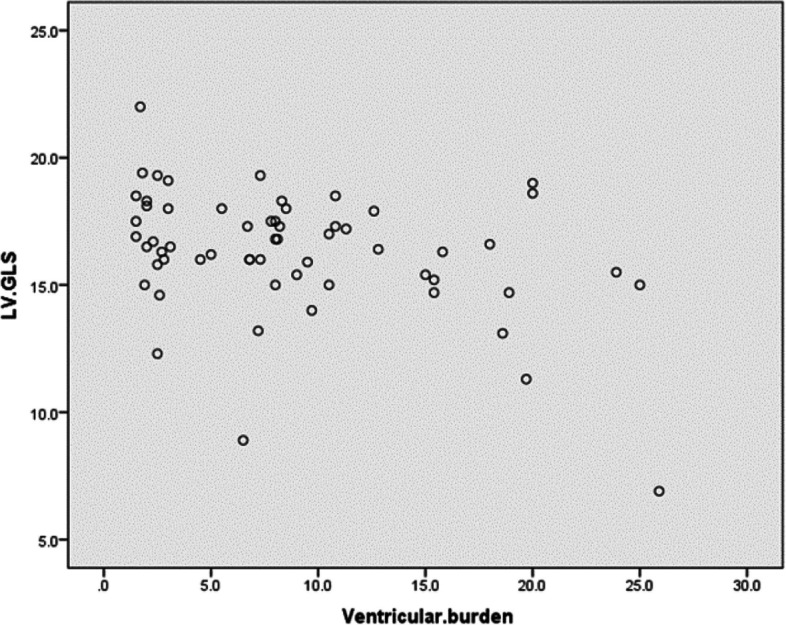
Correlation between ventricular burden and LV-GLS among post COVID symptomatic patients

There were positive correlations between the ventricular burden with LAVI, LV volumes, LV E/AV.E'_4_, LV-MPI, and RV-MPI. While there were negative correlations between ventricular burden with LVEF (bi-plane), MAPSE, TAPSE, LV AV. S^’^_4_, LV-GLS, and RV-GLS (Table 5) and (Fig. 1). Performing multiple linear regression analysis showed that LV-GLS (*p* < 0.001, 95%CI = -1.694 to -0.548), SDNN (*p* < 0.001, 95% CI =  − 0.141 to − 0.063), LF/HF (*p* < 0.003, 95%CI = 1.587 to 3.708), PCFS (*p* < 0.02, 95% CI = 0.429 to 4.368) and CRP (*p* < 0.0001, 95% CI = 0.407 to 1.020) were independently associated with the occurrence of ventricular arrhythmia in symptomatic post-COVID patients.

Table 6 showed the results of binary logistic regression for detecting the association between the occurrence of ventricular arrhythmia (high versus low burden) in symptomatic post COVID-19 patients using demographic, inflammatory biomarkers, echo-Doppler and ambulatory ECG monitoring parameters. The predictors were history of hypertension (*p* < 0.012), elevated CRP (*p* < 0.002), impaired LV-GLS (*p* < 0.001), LV diastolic dysfunction (*p* < 0.001), impaired RV-GLS (*p* < 0.01), reduced TAPSE (*p* < 0.001), abnormal rMSSD (*p* < 0.01) and abnormal SDNN (*p* < 0.0001).

**Table 6:** Binary logistic regression for detecting the association between the occurrence of ventricular arrhythmia using demographic, inflammatory biomarkers, echo-parameters and ambulatory ECG monitoring parameters in symptomatic post COVID-19 patients

Parameters	Odd ratio (95% Confidence interval)	*P*
Demographic parameters		
HTN	17.17 (1.86–158.77)	0.012
Inflammatory biomarkers		
CRP (mg/L) (mean ± SD)	1.69 (1.55–2.07)	0.002
Echo-parameters		
LV-GLS (%)	2.63 (1.63–4.25)	0.001
LV E/ AV.E' _4_	1.56 (1.19–2.04)	0.001
RV-GLS (%)	1.37 (1.08–1.74)	0.01
TAPSE (mm)	1.36 (1.15–1.60)	0.001
Ambulatory ECG monitoring parameters		
rMSSD (ms)	1.13 (1.0–1.27)	0.01
pNN50 (%)	0.87 (0.73–1.03)	0.110
SDNN (ms)	1.04 (1.02–1.06)	0.0001

*Abbreviations*:* HTN* Hypertension, *BMI* Body mass index, *NLR* Neutrophil/lymphocyte ratio, *CRP* C-reactive protein, *LV-GLS* LV global longitudinal strain, *LV E/ AV.E'*_4_ ratio of early diastolic mitral valve velocity/average early diastolic 4-mitral annular velocities by TDI, *RV-GLS* *RV* global longitudinal strain, *TAPSE* Tricuspid annulus plane systolic excursion, *rMSSD* root mean square of the difference between successive normal intervals, *pNN50* the percentage of the number of pairs of consecutive beat-to-beat intervals that differed by 50 ms

**Fig. 2:**
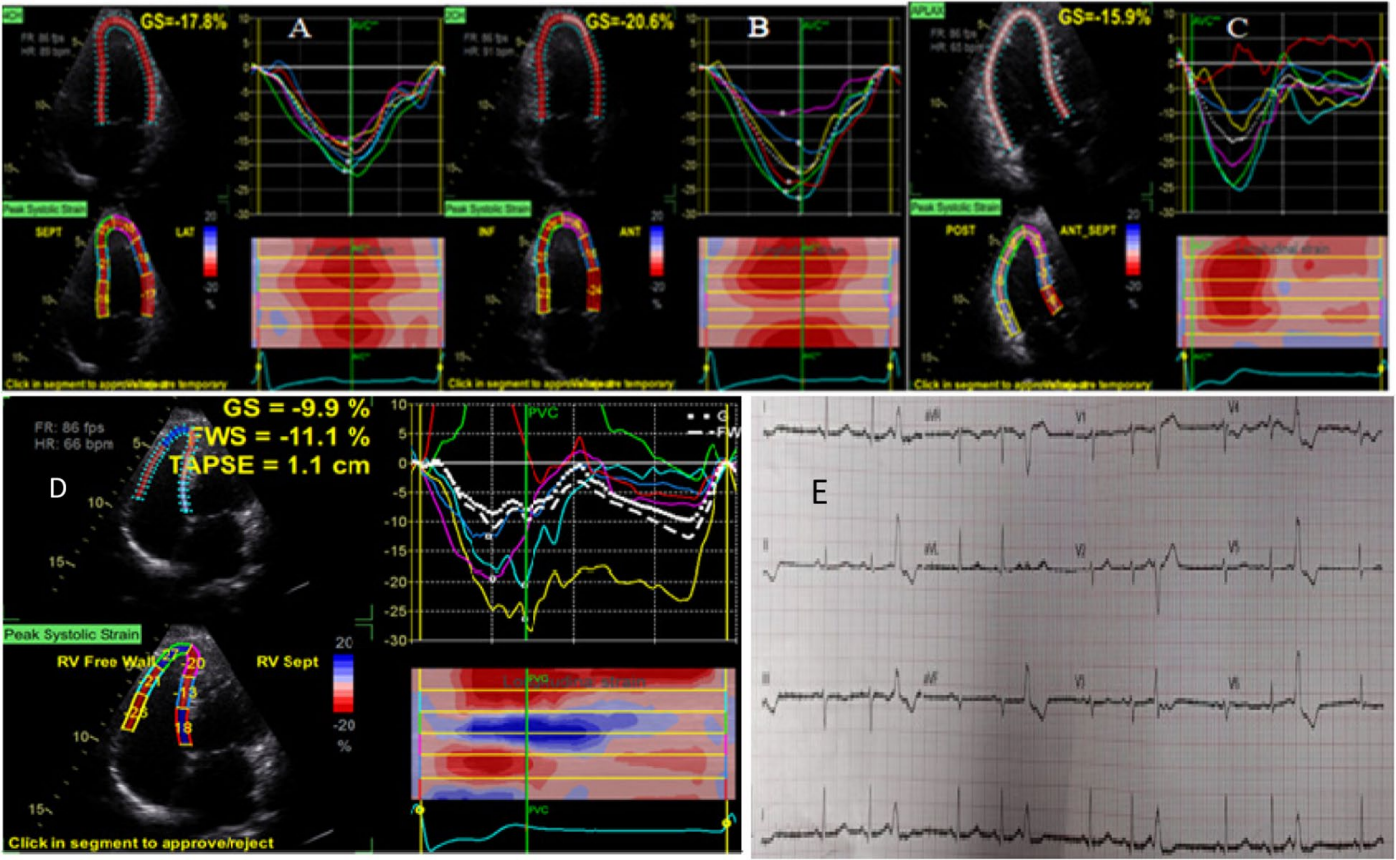
2D- speckle tracking of LV at the apical **A** 4-chamber, **B** 2-chamber, and **C** 3-chamber views, **D** 2D- speckle tracking of RV global and free wall strain; and **E** 12-lead ECG with frequent monomorphic PVCs in a post-COVID patient

Figure 2 showed LV-GLS, RV-GLS, and non-sustained ventricular tachycardia in one of the post-COVID patients.

## Discussion

COVID-19 is considered a systemic infection based on the broad-spectrum clinical signs linked to the involvement of several organs in individuals infected with SARS-CoV-2. Long term effects of COVID-19 are becoming more well identified and have been related to higher morbidity [13]***.***

The term "post-acute sequelae of COVID-19 infection" (PASC) refers to a broad illness with a wide range of symptoms. Patients with PACS experience symptoms following the SARS-CoV-2 infection, which typically last for at least 4 to 12 weeks [14] and [15].

Cardiopulmonary symptoms such as dyspnea, palpitations, decreased physical function, and cardiac arrhythmias might continue for weeks or months in a considerable number of patients (between 10 and 50%). *Øvrebotten *et al*. * [16] indicated that PVCs and non-sustained ventricular tachycardia (18% and 5%, respectively) were the most common types of arrhythmias identified in 27% of the COVID-19 patients 3 to 4 months following hospital release.

The main findings of the current study were (1) patients who had recovered from COVID-19 presented by ventricular arrhythmia had lower functional status and substantial higher levels of inflammatory biomarker, (2) despite that those patients had apparently preserved systolic biventricular function, they demonstrated subclinical impairment of myocardial deformation and lastly (3) abnormal heart rate variability indices were identified in those patients which characterized by increased sympathetic and decreased parasympathetic activity.

In our study, post-COVID patients demonstrated significant impairment of functional status compared to the control group, as detected by higher classes of functional assessment defined by NYHA class and PCFS. In addition, post-COVID patients had considerably lower Hb% and O2 saturation compared to controls, as well as significantly greater WBCs, NLR, and CRP.

Hamdyet al*. *( 17), conducted a multidisciplinary approach and reported that there was significant impairment of functional status among post-COVID patients compared to the control group, and significantly higher levels of the NLR and CRP. For COVID-19 patients, C-reactive protein/albumin ratio (CAR) represented an independent predictive biomarker for in-hospital mortality. CAR may could serve as a biomarker in the initial stages to guide a longer plan of treatment for COVID-19 pneumonia patients ( 18). In patients with COVID-19 disease, NLR ratios can be used to predict the likelihood of mechanical ventilation, ICU admission, and in-hospital death [19].

In the present study, post-COVID patients with higher ventricular burden had a significantly higher resting heart rate compared to those with lower ventricular burden. QTc interval did not differ between both groups. *Lavelle *et al*.* [20] conducted a comparison of a sizable cohort of individuals with and without COVID-19 infection and revealed that COVID-19 infection was associated with a greater QTc interval on the ECG. We hypothesized a residual subclinical post-COVID myocardial inflammatory condition that was accompanied by higher inflammatory markers and persisting electrical abnormalities.

As compared to the control group, post-COVID patients showed a significantly higher level of LV dimensions, volumes, LA diameter, LAVI, E/A, LV E/AV.E'_4_, LV/RV-MPI. In comparison to volunteers, post-COVID patients had significantly lower EF (M-mode and bi-plane), LV AV.S'_4_, LV AV.E'_4_, MAPSE, TAPSE, RV-S'_Lat_ and LV/RV-GLS.

Chaturvediet al*.* [21] showed significant increases in mean LV diastolic dimension, LA volume, and mitral E/E′ were seen at 3-month follow-up in post-COVID patients. Additionally, Özeret al*.* [22] found that during the one-month follow-up following COVID-19 infection, almost one-third of the patients had subclinical LV dysfunction.

Compared to measuring LVEF, studies demonstrate that measuring LV-GLS has a higher sensitivity for detecting mild LV cardiac function impairment [23]***.*** The variation can be explained by the fact that longitudinal strain should primarily reflect myocardial dysfunction, indicating different aspects of the pathophysiology of sepsis-induced cardiac response, whereas LVEF could be affected by the presence of myocardial dysfunction but also be load dependent (hypovolemia, decreased preload, etc.) [24].

It is hypothesized that the cardiac inflammation that might cause tissue fibrosis and stiffness is the cause of the elevated E/E' ratio post-COVID. Long-term effects of this alteration on heart relaxation and diastolic function [25]***.***

Our findings were supported by cross-sectional research byAkkayet al*.* [26] who demonstrated that TAPSE, RV S' by TDI, RV-GLS, and RV-GLS_FW_ strain values were considerably lower in the COVID-19 group with mild severity three months post infection. RV-GLS and RV-GLS_FW_ was inversely associated with acute-phase levels of CRP, NLR, D-dimer, ferritin, and platelet to lymphocyte ratio [26].

There is evidence that the right ventricle is particularly at risk for RV dysfunction following acute COVID-19, which may be explained by the impact of virus-induced lung damage and pulmonary vascular resistance [27] and [23]***.*** These inflammatory and thrombotic markers indicate a pathophysiology that may underlie RV dysfunction, including an interplay of decreased contractility due to cardiac injury and elevated RV afterload [26]***.***

In our research, post-COVID patients had substantially higher HR (minimum and average), larger percentages of tachycardia, ventricular arrhythmia, and higher LF/HF compared to controls. In contrast, post-COVID patients displayed considerably decreased time domain (pNN50, rMSSD, and SDNN) and frequency domain (LF and HF) indices.

Kurtoğluet al*.* [28] observed that when comparing the post-COVID group to the control group, the time-domain parameters of SDNN, rMSSD, and pNN50 were reduced. In comparison to the control group, frequency domain parameters of normalized HF units and HF also reduced in the study group. In agreement, Chistyakovaet al*.* [29] reported that PVCs were seen in individuals who had mild, moderate, or severe COVID-19 infection (in 9.6%, 29.6%, and 57.8% of patients, respectively) three months post-COVID. Additionally, there was a decline in the total HRV SDNN and an increase in the LF/HF ratio in these groups, which most likely denotes a shift in the autonomic balance in the direction of the sympathetic nervous system.

The LF/HF ratio was greater in COVID-19 individuals with lower HRV and increased sympathetic activity as a result of dysautonomia in a comparable study of long-term COVID patients employing 24-h Holter monitoring [30]. Increased sympathetic tone may play a role in the development of ventricular arrhythmia in COVID-19 patients, according to Saha et al. 2022 [31].

In the current study, post-COVID patients with high ventricular burden showed impaired functional status inflammatory biomarker evidenced by NLR and CRP compared to those with lower ventricular burden. Post-COVID patients with high ventricular burden had significantly higher LF/HF in addition to significantly LF and time domain parameters compared to those with lower ventricular burden. The ventricular burden was shown to be closely associated with the functional status, inflammatory markers, LV/LA volumes, LV diastolic function, LV/RV MPI and LF/HF. On the other hand, the ventricular burden had a negative correlation with O2 saturation, HF, LF, pNN50, rMSSD, SDNN, LVEF (bi-plane), MAPSE, TAPSE, LV/RV-GLS. Reduced TAPSE, abnormal rMSSD, LV diastolic dysfunction, impaired LV/RV-GLS and history of hypertension, high CRP were associated with the occurrence of ventricular arrhythmia in symptomatic post-COVID patients.

Arrhythmias in viral myocarditis have been linked to number of possible mechanisms, including membrane lysis-induced electrical imbalance in myocytes, endothelial dysfunction-induced ischemia, and post-inflammatory myocardial scarring. Angiotensin II, which increases cardiac fibrosis and remodels inflammatory cytokines, which alter the function of the cardiac ion channels, can also be linked to reentrant arrhythmias that arise in myocarditis [32]. Myocardial injury in COVID-19 appears to be mostly caused by indirect processes, such as myocardial inflammation, vasculitis, thrombosis, myocardial infarction, or secondary effects of hypoxia, hemodynamic instability, and systemic stress [1] and [33].

In COVID-19 individuals with relatively normal or preserved LVEF, a novel biventricular longitudinal strain may be of special clinical relevance. Additionally, biventricular longitudinal strain during follow-up in COVID-19 patients can provide highly valuable information [34]*.* Because myocardial injury might have unfavourable outcomes, it is crucial to monitor individuals who suffer from it during the acute phase of COVID-19 over the long term. Up to 6.6% of patients in a study of 502 individuals with inflammatory carditis confirmed by biopsy who had their condition prior to the emergence of the COVID-19 pandemic experienced abrupt cardiac mortality or potentially fatal arrhythmia [35]. Patients with ongoing or prior myocarditis showed a higher prevalence of atrial and ventricular arrhythmias [36].

Cannata et al., [37] reported that during 7-month follow-up, 37 patients (34%), who had subclinical myocardial dysfunction—defined as an impairment of LV-GLS—were found to have higher risk of long-term major adverse cardiovascular events (MACE), which included arrhythmic emergencies as a secondary outcome. They recommended that patients who have recovered from COVID-19 pneumonia, speckle-tracking echocardiography is a potentially useful method for optimizing risk stratification [37].

Many investigations have highlighted the correlation between the PVC burden and the decline in LV-GLS [38, 39, 40]. In patients with a high PVC burden, there was a discernible impairment in LV-GLS values. LV-GLS values were found to be negatively linked with higher PVC load and frequency; additionally, a rise in PVC burden was found to be an independent indicator of LV deteriorating-GLS [41]. The decrease in LV-GLS may reflect the cumulative exposure to different cardiovascular risk factors. As a result, LV-GLS may serve as an independent marker for ventricular remodeling, which may serve as a substrate for ventricular arrhythmia. Yoshida et al., proposed that a decreased LV-GLS may serve as a predictive indicator of recurrent ventricular arrhythmias and the subsequent detrimental effects on the cardiovascular system [42].

We suppose that future studies are required to clarify that artificial intelligence (AI) system may be helpful for prediction of post viral ventricular arrhythmia. Successful prediction of the recurrence of paroxysmal AF after catheter ablation was made using an AI-enabled ECG algorithm [43]. AI systems can be used to analyze large amounts of patient data to identify AF risk factors and evaluate the likelihood of developing the condition [44].

### Limitations

The results of the present investigation required confirmation as it was a single-center study with a very small sample size. Additionally, further study is needed to investigate the myocardial deformation compared to healthy individuals with idiopathic ventricular ectopy and comparable arrhythmia burden.

Since our study used workstation software to perform the 2D-STE analysis, the results may not be attainable with other software methods due to the inter-vendor heterogeneity in the 2D-STE parameters.

Not all patients were tested for inflammatory markers (such as D dimer, interleukins, cardiac troponin, and LDH serum ferritin) at the time of acute infection. Therefore, the markers could not be used to evaluate post-COVID syndrome.

Lastly, we excluded vaccine recipients from the current study.

## Conclusions

Low functional status and higher inflammatory biomarkers were observed in post-COVID-19 patients who presented with ventricular arrhythmia. Symptomatic post-COVID patients showed subclinical impairment of myocardial deformation, despite that they seemed to have apparently unaffected biventricular function. Autonomic dysfunction, defined by decreased parasympathetic and elevated sympathetic activity, was observed in symptomatic post-COVID-19 individuals. We suggested using 2D-speckle tracking echocardiography and ambulatory ECG monitoring to enhance risk assessment for patients recovering from COVID-19 infection and exhibiting symptomatic ventricular arrhythmia.

### Supplementary Information

Supplementary Material 1.

## Data Availability

The datasets used and/or analyzed during the current study are available from the corresponding author on reasonable request.

## Abbreviations

CRP: C-reactive protein
EF: Ejection fraction
ESR: Erythrocytic sedimentation rate,
IVSd: Interventricular septum in diastole
LAD: Left axis deviations
LBBB: Left bundle branch block
LV AV. S'_4_: Average systolic 4-sites mitral annular velocities by tissue Doppler imaging
LV AV.E' _4_: Average early diastolic 4-mitral annular velocities by TDI,
LV E/ AV.E' _4_: Ratio of early diastolic mitral valve velocity/average early diastolic 4-mitral annular velocities by TDI
LVEDD: LV end diastolic dimensions
LVEDV: LV end diastolic volume
LVESD: LV end systolic dimensions
LVESV: LV end systolic volume
LV-GLS: Left ventricular longitudinal strain
LV-MPI: Left ventricular myocardial performance
LVOT: Left ventricular outflow tract
LVPWd: LV posterior wall in diastole
MAPSE: Mitral annulus plane systolic excursion
NLR: Neutrophil/lymphocyte ratio,
PASC: Post acute sequlae of COVID-19 infection
PCFS: Post-COVID functional status,
PVCs: Premature ventricular contractions
RAD: Right axis deviation
RBBB: Right bundle branch block
RV-GLS: Right ventricular longitudinal strain
RV-GLS_FW_: RV free wall strain
RV-MPI: Right ventricular myocardial performance
RVOT: Right ventricular outflow tract
RV-S'_Lat_: RV myocardial systolic velocity at the RV lateral wall by TDI
TAPSE: Tricuspid annulus plane systolic excursion
