# Supplementary material for: Clinical utility of ambulatory ECG monitoring and 2D-ventricular strain for evaluation of post-COVID-19 ventricular arrhythmia
Source: BMC Cardiovasc Disord. 2024 Aug 16;24:429. doi: 10.1186/s12872-024-03982-0 (PMC11328462; doi:10.1186/s12872-024-03982-0)
Supplement: Supplementary file 1 — Supplementary Material 1. [file 12872_2024_3982_MOESM1_ESM.docx]

**Highlights**

- Symptomatic post-COVID patients presented by ventricular arrhythmia demonstrated impaired functional status.
- Despite having an apparent preserved biventricular ejection fraction, symptomatic post-COVID patients had subclinical myocardial dysfunction as shown by 2D STE.
- The prevalence of ventricular arrhythmia in post-COVID patients closely linked with increased inflammatory biomarkers and impaired biventricular deformation.
- In 24-hour ambulatory ECG monitoring, symptomatic post-COVID patients showed autonomic dysfunction, which is characterized by low parasympathetic activity and high sympathetic activity.
